# Motion Coupling at the Cervical Vertebral Joints in the Horse—An Ex Vivo Study Using Bone-Anchored Markers

**DOI:** 10.3390/ani15152259

**Published:** 2025-08-01

**Authors:** Katharina Bosch, Rebeka R. Zsoldos, Astrid Hartig, Theresia Licka

**Affiliations:** 1 Clinical Department for Small Animals and Horses, University of Veterinary Medicine Vienna, 1210 Vienna, Austriatheresia.licka@vetmeduni.ac.at (T.L.); 2 Department of Biosystems and Technology, Swedish University of Agricultural Sciences, 23422 Alnarp, Sweden; 3 School of Agriculture and Food Sustainability, The University of Queensland, Gatton, QLD 4343, Australia; 4 Royal (Dick) School of Veterinary Studies, University of Edinburgh, Edinburgh EH8 9YL, UK

**Keywords:** equine, cervical, spine, kinematics, redundancy, vertebral motion, yaw, pitch, roll

## Abstract

The influence of soft tissue structures, including ligaments spanning one or more intervertebral junctions and the nuchal ligament, on motion of the equine cervical joints remains unclear. The present study addressed this using four post-mortem horse specimens extending from head to withers with all ligaments intact. Three-dimensional kinematics was obtained from markers on the head and bone-anchored markers on each cervical and the first thoracic vertebra during rotation, lateral bending, flexion and extension of the whole head, and neck segment. Yaw, pitch, and roll angles in 8 cervical joints (total 32) were calculated. Flexion and extension were expressed mainly as pitch in 27 and 22 joints, respectively. Rotation appeared as predominantly roll in 13 joints, whereas lateral bending was represented as predominantly yaw in 1 and as roll or pitch in all other joints. Significant correlations between yaw, pitch, and roll were observed at individual cervical joints in 97% of all measurements, with the atlanto-occipital joint showing complete (100%) correlation. Most non-significant correlations occurred at the C5–C6 joint, while C6–C7 exhibited significantly lower correlation coefficients compared to other levels. The overall movement of the head and neck is not replicated at individual cervical joint levels and should be considered when evaluating equine necks in vivo.

## Simple Summary

The horse’s neck is highly flexible and vital for balance, vision, and breathing. Many horses develop neck problems, but detailed motion of each neck joint is still poorly understood. Most past research removed the neck’s ligaments to make measurements easier, even though these tissues help the neck store and release energy during trotting or cantering. The present study looked at how different joint movements are linked with the ligaments still in place. Using donated horse bodies placed upright in a wooden frame, markers were fixed onto the head, and onto small pins in each neck bone and the first bone of the chest. The bones were then tracked as the head was turned, nodded, and tilted by hand. A strong link was found between movements; when the head moves to the side, most neck joints also move sideways. Nodding motion was strongest at the “yes joint” between the head and the first neck bone and turning was strongest at the “no joint” between the first and the second neck bone. No joint in the neck moved independently. Understanding how the horse neck moves can help improve horse training and treatments for neck problems.

## 1. Introduction

The equine cervical spine is a complex system within the equine locomotor system and, showing more mobility than the thoracolumbar spine, it has long been the topic of research [1]. Similar to most other mammals [2], the horse has seven cervical vertebrae (C1–C7), of which the first two, atlas and axis, differ anatomically in shape and function from the more caudal vertebrae. The occiput has large oval-shaped condyles that extend deep into the atlas. The atlas, shaped like a ring, forms almost saddle-shaped facets with the dens of the axis, which is the longest cervical vertebra. From the axis caudally, vertebral motion at intervertebral junctions is allowed by synovial zygapophyseal (facet) joints and intervertebral discs. The gradual change in anatomy throughout the cervical spine is reflected in the compact vertebral bodies and thick intervertebral discs of C6 and C7 that absorb the larger compressive forces exerted there [3]. This makes them slightly more similar to the thoracic vertebrae than the more cranial cervical vertebrae. Furthermore, the last cervical vertebra, C7, connects with the first pair of ribs as part of the relatively stable cervicothoracic junction [4,5].

With the head and neck accounting for roughly 10% of body mass [6], their high mobility supports essential activities like feeding and balance during locomotion [7]. The cervical spine is anatomically exposed and biomechanically active, making muscular stabilization vital, especially in aging horses where muscle function declines [8]. Degenerative changes in the neck are more often due to mobility and forces exerted than to trauma, with common conditions including facet joint osteoarthritis, nerve compression, and soft tissue injuries [9]. Passive structures such as ligaments, joint capsules, and fasciae help limit excessive motion and protect the spinal cord [10,11,12]. The nuchal ligament, particularly well developed in horses, contributes to this stability through its funicular and lamellar parts connecting the thoracic spine to the occiput and cervical vertebrae [13,14].

For the present study, we define the global coordinate system (yaw, pitch, and roll) for the horse relative to its anatomical axes at normal posture [15,16]. Specifically, yaw corresponds to rotation around the dorsoventral axis (e.g., bending the spine sideways), pitch around the latero-lateral axis (e.g., flexing and extending the spine), and roll around the craniocaudal axis (e.g., body rotation). This is different to birds and humans as well as mammals with an upright vertebral column during normal posture, where yaw is rotation of the head against the thorax and roll is lateral bending the head towards the shoulder [15,17,18]. For the remainder of this paper, human and other upright spine motions are identified using the prefix “H” followed by the corresponding quadruped movement terminology (e.g., H_yaw = roll). In the present study, the term “cervical vertebral joint (CVJ)” is used for all articulations involving the cervical vertebrae, including atlanto-occipital (CVJ1), atlanto-axial (CVJ2), other cervical intervertebral (CVJ3–7), and cervicothoracic (CVJ8) joints.

The relationship between yaw, pitch, and roll at the vertebral joints of the head and neck has previously been examined in vitro and in vivo in humans [18,19,20], as well as in sloths [21] and owls [16]. In human patients, magnetic resonance imaging (MRI) showed coupled motions of the upper cervical spine both during lateral bending [18] and during rotational movements [19]. This supported results of earlier in vitro studies [20], indicating the value of initial in vitro investigations.

For the horse, investigations of the movement of cervical vertebrae have been performed both in vitro [22,23,24,25] and in vivo [26,27]. In vitro studies allow for the use of bone-anchored markers and 3D dynamic imaging, and, for practical reasons, studies mostly use isolated cervical spines without muscles and elastic soft tissues. In 1989, Clayton and Townsend [22] used optical motion capture to examine the isolated cervical spine without muscles and large ligaments and found coupling of lateral bending and rotation between C3 and T1. Furthermore, motions for CVJ1 and CVJ2 were shown to be specialised, as expected. Motion in the mid-cervical spine increased from cranial to caudal until CVJ7 but was smaller at CVJ8. Schulze et al. assessed the impact of cervical joint fusion, based on computed tomography (CT) scans and revealed that motion patterns in equine neck specimens were consistent with those reported in a prior in vitro investigation before surgical fixation [23].

In equine in vivo studies, the movement of the head and cervical vertebrae was measured using optical motion capture with markers placed on the skin, with the advantage of including all soft tissues but the disadvantage of skin movement against the vertebrae [26,27]. Zsoldos et al. [27] developed a kinematic model using musculoskeletal modelling software based on horses moving on a treadmill, which revealed that CVJ6 had the smallest range of motion across all movement directions. The largest flexion–extension motion was observed at CVJ4, the greatest axial rotation at CVJ2, and maximum lateral bending at CVJ8. In contrast to earlier in vitro findings [22], the greatest head and spine movement during dynamic mobilization exercises in live horses, involving cervical lateral bending, occurred at the cervicothoracic region (C6–T6) and less mobility was seen in the mid-cervical area [26]. This apparent discrepancy may reflect the stabilising influence of musculature activity as well as of the passive structures in vivo, suggesting that functional movement is reduced compared to the anatomical movement possibilities of the CVJs.

In horses, an in vitro study showed that the atlanto-occipital (CVJ1) joint permits approximately 10° of flexion, 15° of extension, and 8–10° of lateral bending per side, with minimal axial rotation [22]. The atlanto-axial (CVJ2) joint accounts for the majority of cervical rotation (~25–30° per side), while lateral bending peaks in the mid-cervical region at C5–C6 (6–8°) and C6–C7 (5–7°) before decreasing toward the cervicothoracic junction. This was shown in vitro and in the kinematic model based on live horses moving [22,27].

Motor redundancy, or better motor abundance [28], a state in which several combinations of locomotor elements can be used to perform a specific motor task, has been described for human arm movement [29,30], but it is also a basic principle of movement widely considered as an evolutionary advantage [31]. It provides robustness and adaptability in movement, including the ability to compensate for injury, fatigue, or environmental perturbations. Asymptomatic human individuals exhibit correlated but variable and unpredictable spinal segment motions of the thoracic and lumbar spine during daily activities, with inherent redundancy or abundance and significant between-subject variability, as determined by kinematic measurements and inertial sensors [32]. While existing human studies have focused on thoracic and lumbar spinal regions, the concept of motor redundancy or abundance is equally relevant to other spinal regions and other species.

Despite the horse being primarily bred and used for its locomotion, more so than other domesticated ungulates such as cattle, sheep, or goats, motor abundance or redundancy has not yet been demonstrated in equine movement. The aim of this in vitro study was to investigate how yaw, pitch, and roll are combined at the equine CVJs during passively induced movements of the head and neck segment of cadavers, and to investigate potential differences in how these movements are expressed at the different levels of the cervical spine in individuals.

## 2. Materials and Methods

### 2.1. Specimens

This study evaluates a subset of data obtained for investigation of surgical procedures at the CVJs. In the present study, four adult equids—three horses and one end-sized pony—of various breeds and ages were used. They were euthanised with the owner’s consent at the University for Veterinary Medicine in Vienna for reasons unrelated to the cervical spine or neurological symptoms (Table 1).

No abnormalities related to the cervical spine had been noted in the medical history or during the clinical examination of these horses. Owners had given their consent for the use of animal material in scientific studies. The head, neck, thoracic spine, ribcage (excluding thoracic organs), and sternum were removed as a single unit from the euthanised animal. The skin, subcutaneous tissues, trachea, oesophagus, and all muscles superficial to the M. longus colli, Mm. multifidi, Mm. interspinales, Mm. rotatores, and Mm. intertransversarii cervicis were removed. A full list of muscles removed is provided in Appendix A.

**Figure 1:**
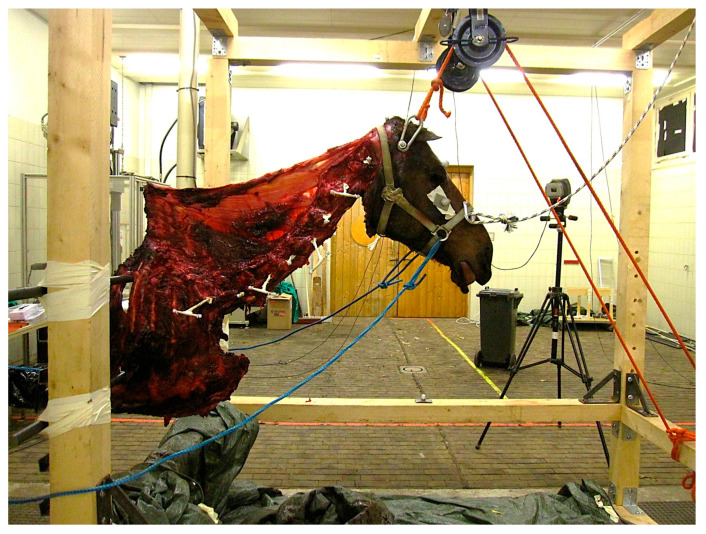
Original lab setting of a head and neck segment in a neutral position, secured in the measurement frame with steel rods between the fifth and sixth rib. Ropes running through a pulley system are attached to a transverse bolt through the base of the skull to suspend the head. T-bar pins are visible in each cervical and the first thoracic vertebra, the side of the T piece and the single points are alternating. On all three end points of the T-bar pins, reflective, spherical kinematic markers are attached. The kinematic markers on the right facial crest and on the frontal bone are visible. Ropes attached to the headcollar were used to achieve head and neck movements. The specimen is of horse L, part of the larger study group.

All spinal ligaments and joint capsules, as well as both the lamellar and the funicular parts of the nuchal ligament, were left intact (Figure 1). The thoracic spine, the rib cage, and the sternum were docked at the level of T6, and the specimens were frozen at −20° Celsius for at least 1 week and a maximum of 5 weeks. Prior to data collection, specimens were stored at a room temperature of about 15 °C for 24 to 48 h until fully thawed.

After completion of data acquisition, the necks were dissected (K.B.) and evaluated for gross pathological abnormalities.

### 2.2. Mounting of the Specimen and Measurements

**Figure 2:**
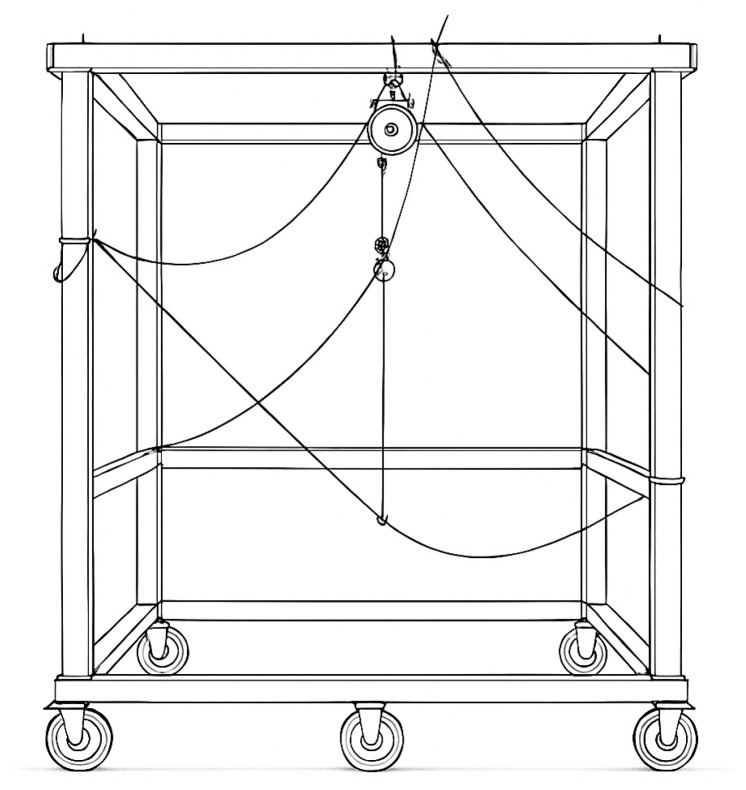
Stylised sketch of the wood frame 1.80 m long, 1.5 m wide, and 1.9 m high used for supporting the specimens in an upright position. The two pulleys at the top secured the steel bolt inserted through the skull. Ropes attached to the head collar were used to manually achieve the movement of the head.

A steel bolt was inserted latero-laterally through the base of the skull and the specimen was lifted using a forklift. Two pulleys secured the steel bolt at the top of a large wood frame 1.80 m long, 1.5 m wide, and 1.9 m high (Figure 2) in a neutral head and neck position, as two solid steel bars inserted between the fifth and sixth rib were placed into steel U-profiles additionally secured with textile tape at the back of the wooden frame. Only once the specimen was safely attached was the forklift removed.

**Figure 3:**
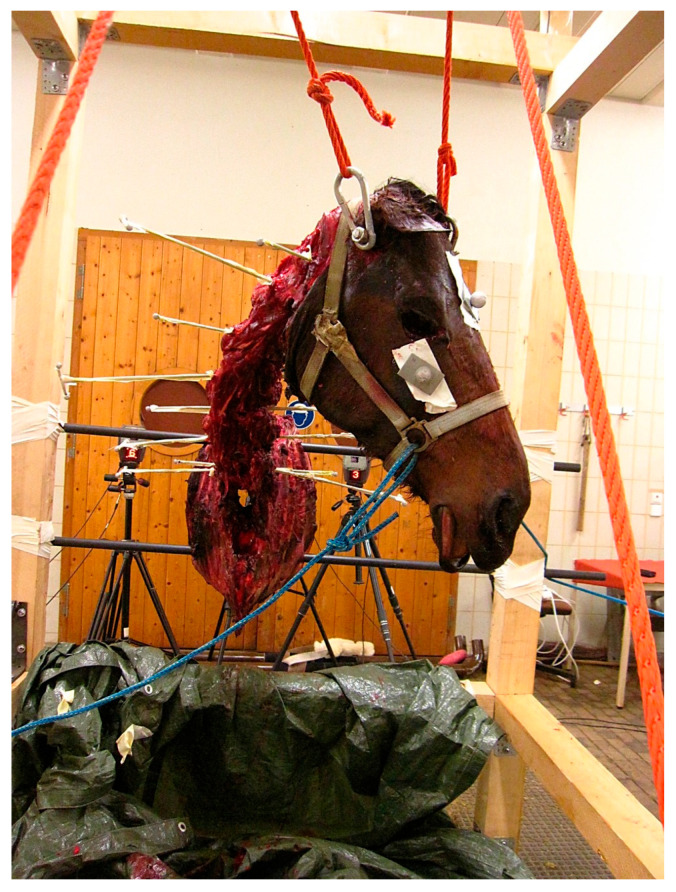
Original lab setting of a head and neck segment during lateral bending to the left, secured in the measurement frame with steel rods between the fifth and sixth rib. Ropes running through a pulley system are attached to a transverse bolt through the base of the skull to suspend the head. T-bar pins are visible in each cervical and the first thoracic vertebra, the side of the T piece and the single points are alternating. On all three end points of the T-bar pins, reflective, spherical kinematic markers are attached. The kinematic markers on the right facial crest and on the frontal bone are visible. Ropes attached to the headcollar were used to achieve head and neck movements. The specimen is of horse L, part of the larger study group.

Thirty reflective markers attached with double-sided adhesive tape were used for kinematic tracing. Three spherical markers of 14 mm diameter were placed on the bony landmarks of the head (facial crest left and right, frontal bone) and three on the wooden frame (left, right, middle). The atlas was drilled latero-laterally through its centre just ventral to the spinal canal, and all other vertebrae (C2–T1) were drilled latero-laterally through the mid-portion of the vertebral body. Threaded T-bar pins (length 50 cm, T bar 8 cm) were inserted starting with the T-bar piece on the left in C1 and alternating between the left and right side in the following vertebrae (Figure 3). On each of the three points of the T-bar spherical, reflective markers of 9 mm diameter were secured. A tightly fitting, non-reflective head collar was used for manual movement of the head with the help of ropes.

All movements were performed by the same person (K.B.) in the same order and manner for all horses, aiming to stay as close as possible to the natural physiological movement. The movement was visually monitored to ensure a slow, steady speed and a range well within the estimated in vivo maximum for a horse of that size.

Three-dimensional kinematic data were collected using ten high-speed digital infrared cameras (Santa Rosa EVaRT, version 5.0.4; Eagle Digital Real-Time System; Motion Analysis Corporation, Santa Rosa, CA, USA) recording at 120 Hz. Data were captured and collected using a personal computer and motion-analysis program (EVaRT, version 5.0.4; Motion Analysis Corporation, Santa Rosa, CA, USA). The cameras were calibrated before each data collection using an L-shaped calibration frame and a calibration wand.

Data were collected for 6 defined movements for each specimen: flexion, extension, lateral bending left, lateral bending right, axial rotation left (defined as the nose turning to the left), and axial rotation right. Each of these overall movements of the head and neck segment was achieved across two and three repetitions during a 10 s time frame and that time frame repeated three times for each overall movement (3 × 10 s per movement, 180 s for all movements). Movements were generated by pulling on ropes attached to the head collar, with the pulley system suspending the skull allowing adequate flexibility.

### 2.3. Data Processing and Analysis

Marker movements were determined using kinematic processing software (Cortex-64 7.0.0., Motion Analysis Corporation, Santa Rosa, CA, USA). Kinematic data were then smoothed with a Butterworth low-pass filter (cut-off frequency 6 Hz), and further processed in Excel (Microsoft Office 2016, Microsoft Corporation, Redmond, WA, USA).

Between the planes defined by 3 markers per segment, Euler angles and quaternions identifying yaw, pitch, and roll were calculated for each CVJ from the atlanto-occipital joint (CVJ1) to the cervicothoracic junction (CVJ8). The maximum and minimum angle as well as the range of motion (ROM) were determined for each movement and mean values per horse and movement were taken forward. Similarly, in the three axes x, y, and z, absolute displacement of segments was calculated.

Statistical data analysis was carried out using SPSS (IBM SPSS Statistics 24, IBM, Armonk, NY, USA). To compare overall motion patterns across movement types, values were first averaged across all cervical segments for each horse. Two-factored Friedman tests and Kendall coefficients were used to compare movements of the left and right side. A Friedman test was then used to assess differences between the four horses across the four movement directions. Statistical analysis of inter-horse differences of angular movement was conducted using the Friedman test applied separately for each movement type, lateral bending (LB), axial rotation (AR), flexion (Flex), and extension (Ext), to evaluate differences among the four horses across the nine segments and eight cervical joints. Where the Friedman test revealed significant differences, pairwise Wilcoxon signed-rank tests with Bonferroni correction were used for post hoc comparisons. For angles, tests were conducted using average motion values per joint derived from the three rotational axes (yaw, pitch, and roll). For each CVJ of each horse, the coupling of yaw, pitch, and roll was calculated using the two-way Pearson correlation coefficients (PCC) for the six overall movements. Trend fitting of PCCs over the CVJs was performed using both linear and exponential functions to determine the best fit. Statistical significance was defined at *p* < 0.05.

## 3. Results

At post measurement dissection, no gross pathologies of the head and neck segments were noted. No significant difference in the overall lateral bending movement of the head and neck segment towards left and right and in the overall rotation movement clockwise and anticlockwise was found. Therefore, both directions are presented together in the remainder of the paper.

When motion values were averaged across all segments, and horses were compared across the four movement types (axial rotation, extension, flexion, lateral bending), the Friedman test showed no significant differences (*p* = 0.212). This suggests that when looking at movement direction as the main variable, overall motion patterns were comparable across the four horses.

### 3.1. Range of Segmental Displacement at the Four Overall Movements

**Figure 4:**
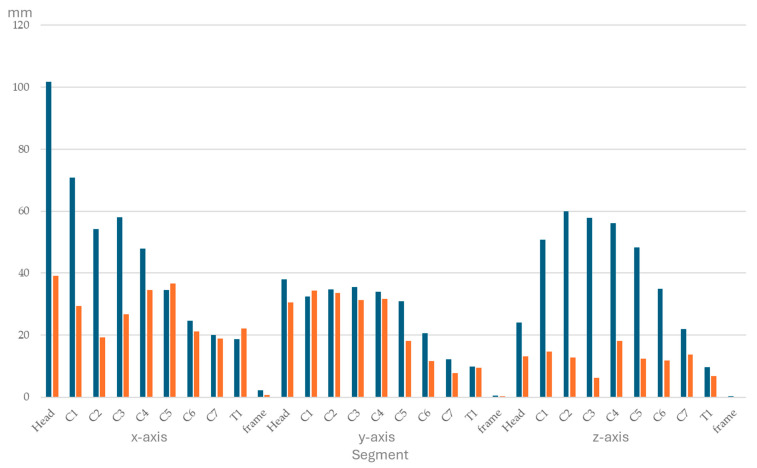
Range of segmental displacement (in mm) achieved during flexion of the head and neck segment in four horses is presented as median values (blue bars) with interquartile range values (orange bars) from the mean of the three markers per segment per horse and for the frame. Segments are head, cervical vertebrae (C1–C7), and the first thoracic vertebra (T1). Data for the *x*-, *y*-, and *z*-axes are depicted on the left, in the middle, and on the right, respectively.

**Figure 5:**
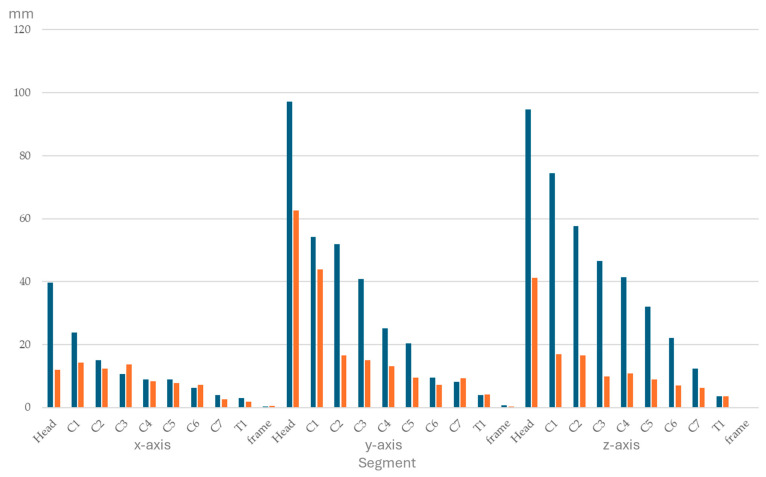
Range of segmental displacement (in mm) achieved during extension of the head and neck segment in four horses is presented as median values (blue bars) with interquartile range values (orange bars) from the mean of the three markers per segment per horse and for the frame. Segments are head, cervical vertebrae (C1–C7), and the first thoracic vertebra (T1). Data for the *x*-, *y*-, and *z*-axes are depicted on the left, in the middle, and on the right, respectively.

**Figure 6:**
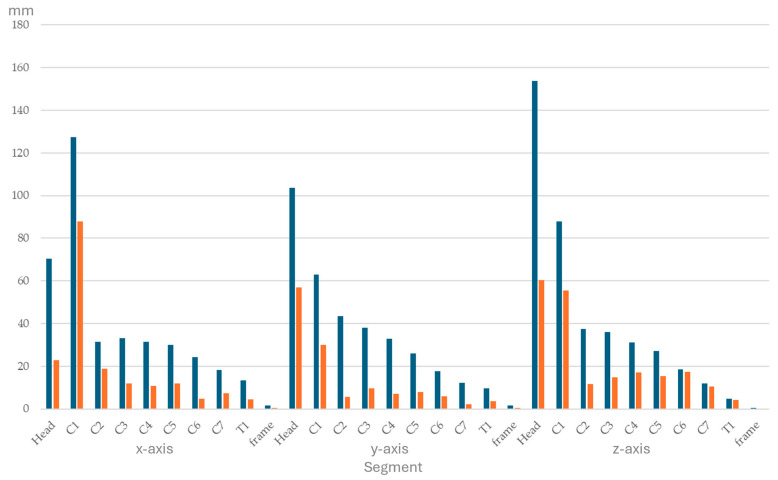
Range of segmental displacement (in mm) achieved during axial rotation of the head and neck segment in four horses is presented as median values (blue bars) with interquartile range values (orange bars) from the mean of the three markers per segment per horse and for the frame. Segments are head, cervical vertebrae (C1–C7), and the first thoracic vertebra (T1). Data for the *x*-, *y*-, and *z*-axes are depicted on the left, in the middle, and on the right, respectively.

**Figure 7:**
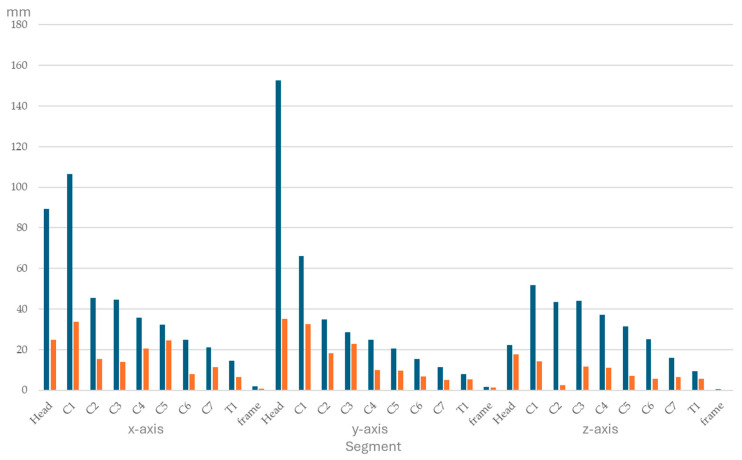
Range of segmental displacement (in mm) achieved during lateral bending of the head and neck segment in four horses is presented as median values (blue bars) with interquartile range values (orange bars) from the mean of the three markers per segment per horse and for the frame. Segments are head, cervical vertebrae (C1–C7), and the first thoracic vertebra (T1). Data for the *x*-, *y*-, and *z*-axes are depicted on the left, in the middle, and on the right, respectively.

Ranges of segmental displacement data for flexion, extension, axial rotation, and lateral bending at the 10 segments are presented in Figure 4, Figure 5, Figure 6 and Figure 7, respectively. Segmental displacement decreased from CVJ1 to CVJ8 exponentially for extension (y = 1.474 × 10^−0.445^x, R^2^ = 0.542), axial rotation (y = 176.9 × 10^−0.383^x, R^2^ = 0.6655), and lateral bending (y = 148.2 × 10^−0.343^x, R^2^ = 0.5413), and linearly for flexion (y = −5.8554x + 67.743, R^2^ = 0.4063).

### 3.2. Overall Head and Neck Movement Angles

**Figure 8:**
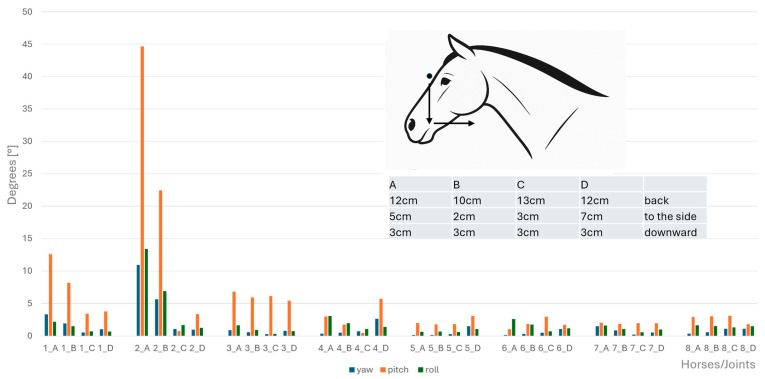
Angular motion (x-axis, in degrees) for yaw (blue bars), pitch (orange bars), and roll (green bars) at the eight cervical intersegmental junctions (1: atlanto-occipital joint to 8: cervicothoracic junction) are shown for each of the four horses (A–D), based on the intraindividual mean values of flexion of the head–neck segment. Corresponding head displacements (in cm) from the neutral position along the three spatial axes are provided in the table at the top left of the figure and illustrated in the lateral view of the horse.

**Figure 9:**
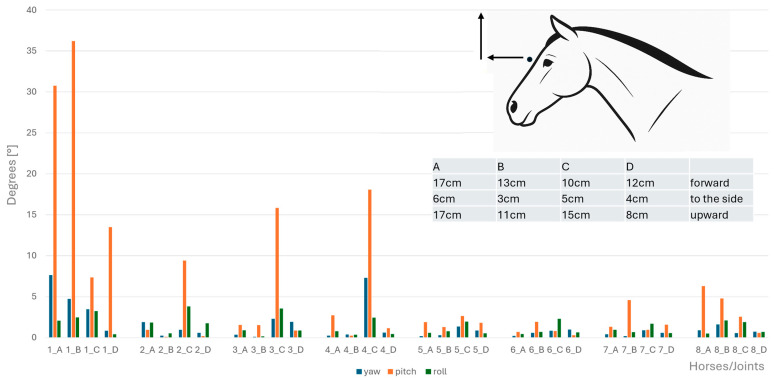
Angular motion (x-axis, in degrees) for yaw (blue bars), pitch (orange bars), and roll (green bars) at the eight cervical intersegmental junctions (1: atlanto-occipital joint to 8: cervicothoracic junction) are shown for each of the four horses (A–D), based on the intraindividual mean values of extension of the head–neck segment. Corresponding head displacements (in cm) from the neutral position along the three spatial axes are provided in the table at the top left of the figure and illustrated in the lateral view of the horse.

**Figure 10:**
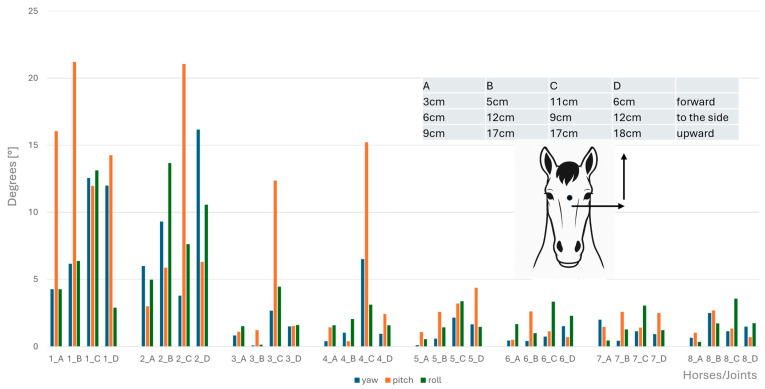
Angular motion (x-axis, in degrees) for yaw (blue bars), pitch (orange bars), and roll (green bars) at the eight cervical intersegmental junctions (1: atlanto-occipital joint to 8: cervicothoracic junction) are shown for each of the four horses (A–D), based on the intraindividual mean values of clockwise and anticlockwise axial rotation of the head–neck segment. Corresponding head displacements (in cm) from the neutral position along the three spatial axes are provided in the table at the top left of the figure and illustrated in the lateral view of the horse.

**Figure 11:**
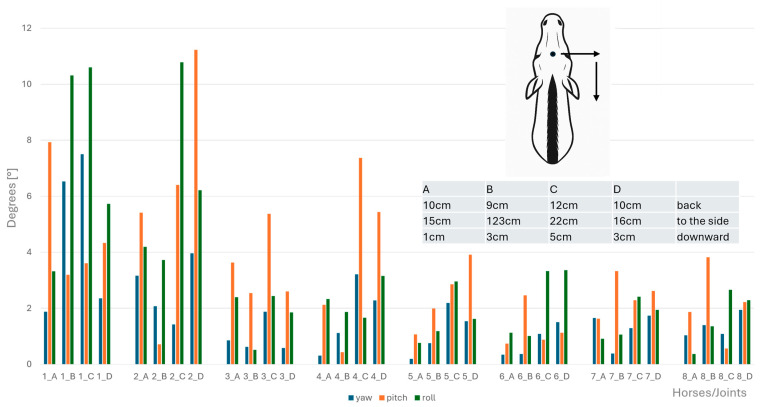
Angular motion (x-axis, in degrees) for yaw (blue bars), pitch (orange bars), and roll (green bars) at the eight cervical intersegmental junctions (1: atlanto-occipital joint to 8: cervicothoracic junction) are shown for each of the four horses (A–D), based on the intraindividual mean values of left- and right lateral bending of the head–neck segment. Corresponding head displacements (in cm) from the neutral position along the three spatial axes are provided in the table at the top left of the figure and illustrated in the lateral view of the horse.

Angular motion at the CVJs during flexion, extension, axial rotation, and lateral bending were significantly different between horses; specifically, a significant difference between Horse B and Horse D for both lateral bending (*p* = 0.047) and axial rotation (*p* = 0.047) was established. No other pairwise comparisons reached significance after correction. Therefore, results of angular motion are presented for individual horses in Figure 8, Figure 9, Figure 10 and Figure 11. These figures also illustrate the corresponding head displacements from the neutral position using relevant anatomical views (lateral, frontal, and dorsal).

### 3.3. Correlation Between Yaw, Pitch, and Roll

**Figure 12:**
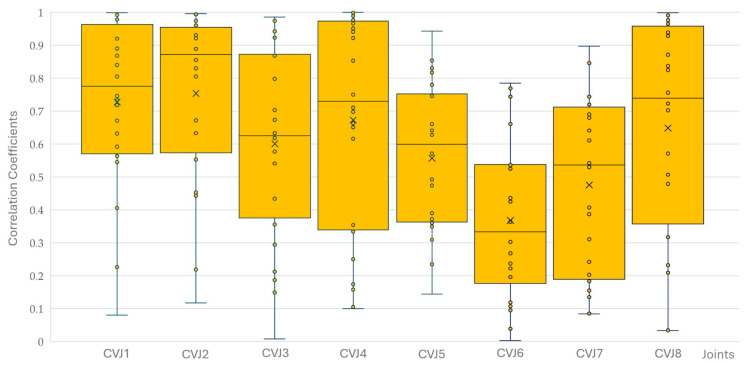
Pearson correlation coefficients between yaw and pitch motions during flexion, extension, axial rotation, and lateral bending of the head and neck segment of four horses are shown as box plots of the resulting 16 values for each of the eight cervical intersegmental junctions (CVJ1: atlanto-occipital joint to CVJ8: cervicothoracic junction). All individual data points are plotted as small circles (°). The orange boxes represent the interquartile range (IQR), with the horizontal line inside each box indicating the median. Whiskers extend to the most extreme data points within 1.5 times the IQR. Outliers beyond this range are marked with small circles (°), and the mean value for each group is indicated by a cross (×).

**Figure 13:**
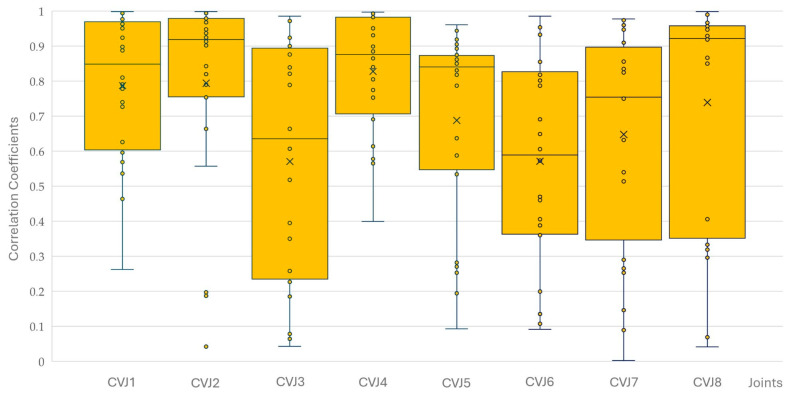
Pearson correlation coefficients between yaw and roll motions during flexion, extension, axial rotation, and lateral bending of the head and neck segment of four horses are shown as box plots of the resulting 16 values for each of the eight cervical intersegmental junctions (CVJ1: atlanto-occipital joint to CVJ8: cervicothoracic junction). All individual data points are plotted as small circles (°). The orange boxes represent the interquartile range (IQR), with the horizontal line inside each box indicating the median. Whiskers extend to the most extreme data points within 1.5 times the IQR. Outliers beyond this range are marked with small circles (°), and the mean value for each group is indicated by a cross (×).

**Figure 14:**
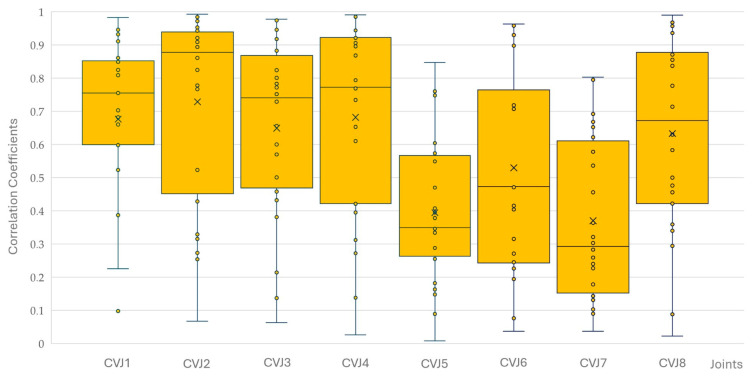
Pearson correlation coefficients between pitch and roll motions during flexion, extension, axial rotation, and lateral bending of the head and neck segment of four horses are shown as box plots of the resulting 16 values for each of the eight cervical intersegmental junctions (CVJ1: atlanto-occipital joint to CVJ8: cervicothoracic junction). All individual data points are plotted as small circles (°). The orange boxes represent the interquartile range (IQR), with the horizontal line inside each box indicating the median. Whiskers extend to the most extreme data points within 1.5 times the IQR. Outliers beyond this range are marked with small circles (°), and the mean value for each group is indicated by a cross (×).

There were no significant differences between the four horses for yaw, pitch, and roll correlations at the eight CVJs. The correlation coefficients for yaw, pitch, and roll decreased significantly from CVJ1 to CVJ8 (PCC −0.204; *p* < 0.001). Of 576 correlations calculated (six movements of four horses with three combinations of yaw/pitch/roll at the eight CVJs), 559 were significantly correlated; of those, 553 were highly significant. Most non-significant correlations were found between yaw and pitch (*n* = 8), followed by pitch and roll (*n* = 5), and yaw and roll (*n* = 4). Non-significant correlations were most observed at CVJ6 (*n* = 5) and CVJ8 (*n* = 4), while all correlations at CVJ1 reached statistical significance. Figure 12, Figure 13 and Figure 14 present Pearson correlation coefficients between yaw and pitch (Figure 12), yaw and roll (Figure 13), and pitch and roll (Figure 14) during the four movement types, across the eight cervical intersegmental junctions. These are displayed as box plots, showing variability across all trials.

## 4. Discussion

The present study documents the coupling of yaw, pitch, and roll in the intersegmental junctions of isolated head and neck segments, with the ligamentum nuchae intact, of horses during overall movements that were roughly within the middle third of the maximum movements achieved by live horses [26]. To the best of the authors’ knowledge, this is the first presentation of this basic biomechanic relationship in this species.

Understanding how the equine neck moves is essential for effective training and treatment, as it plays a central role in balance, locomotion, and overall biomechanics. The neck acts as a dynamic balancing mechanism, integrating visual, vestibular, and proprioceptive input, while its movement influences both the trunk and limbs through interconnected bony, ligamentous, and muscular structures [33]. Research has shown that neck position, particularly when altered by riders or training aids, can significantly affect stride length and back flexibility, with hyper-flexed or highly restricted positions raising welfare concerns and potentially compromising performance [34,35]. Accurate assessment of cervical motion is, therefore, critical for diagnosing and treating neck pain or dysfunction, enabling the development of safer, more effective training methods and targeted rehabilitation strategies that support long-term health and performance [10].

Anatomical distinctions along the cervical column are reflected in the kinematic findings, which revealed a progressive reduction in motion at the cervical vertebral joints (CVJs) from cranial to caudal. This shows the morphological shift from flexible, coupling-facilitating cranial vertebrae, over the mid-cervical vertebrae to the more rigid and load-bearing caudal segments, supporting the functional specialization along the cervical spine. The first two CVJs showed considerably larger motions than CVJs 3–6, where vertebrae have the typical architecture of the equine cervical spine. In vertebrae C3–C5, the flat articular facets on the cranial articular processes are oriented dorsally, medially, and cranially, and in the opposite direction on the caudal processes. In contrast, in the caudal cervical vertebrae (C6 and C7), the orientation of the facet joints changes such that the articular surface of the cranial articular processes faces more dorsally, while that of the caudal articular processes faces more ventrally.

Pitch was the largest motion at CVJ7 and CVJ8 in two thirds of the overall motions in the present study. Despite the assumption of CVJ8 being structurally stable [4] with the first pair of ribs and surrounding musculature, anchoring the cervicothoracic junction, motion results and coupling were very similar to CVJ7. Even though cranial ribs, cranial thoracic vertebrae, and the cranial part of the sternum were part of the specimens investigated, the lack of musculature may account for this difference. A motion reduction from cranial to caudal in vitro has been published before [22], but it seems that in the live horse, the influence of the whole body on the motion at the caudal CVJs is more pronounced [26,27] and allows for a wider range of motion in this area.

A study performed on cadaveric specimens of foxhounds, using four levels of the cervical spine devoid of all tissues, established no difference between flexion and extension. This was not the case for the present study, where the overall movement of flexion was much larger than that of extension. In the canine specimens, lateral bending tended to be greater in the cranial spine, the opposite was found for the equine specimens in the present study. In addition, axial rotation was much smaller in the caudal cervical spine of horses than dogs [36].

One of the key findings was that the overall movement of the head relative to the thorax was achieved through different motions at the individual CVJs. This suggests that in the equine cervical spine, movement redundancy or abundance also plays a role, allowing a variation of combinations of joints and motions at these joints to produce the same overall movement. This concept of movement redundancy or abundance is well-documented in human motor control research [28,29,30]. Rather than a consistent distribution of the overall movement of the body or body parts onto motions at specific joints, joints contribute differently in individuals, as is clearly seen in the variations in yaw, pitch, and roll angles at the cervical vertebral joints and the variations in correlations between them, reflecting both intra- and interindividual variability in movement strategies [30,31].

The large degree of coupling observed in all necks studied was consistent across overall movements. Even the first two CVJs, which are anatomically predisposed for pitch (CVJ1) and roll (CVJ2), showed high coupling with other motions. At CVJ1, pitch dominated in axial rotation, flexion, and extension, while roll was dominant during lateral bending. At CVJ2, motion was equally distributed between pitch and roll, suggesting a more versatile biomechanical role than previously described [4,5,37]. Thus, dysfunction at CVJs 1 and 2, as is the case in foals with occipito-atlanto-axial malformation [38] or after trauma [39,40], should be carefully assessed in clinical imaging of osseous and soft tissues and targeted in treatment and rehabilitation. Complete consistency in correlated motions across all directions was found for CVJ1, underscoring its central role in coordinated neck movement and its potential impact in both cervical disease management and surgical intervention planning.

The present study shows that in vitro lateral bending of the head and neck segment is achieved by pitch and roll motions at individual CVJs and the combination of these motions translates to the overall lateral movement achieved. The underrepresentation of lateral CVJ motions during lateral bending has been discussed previously [20] in an in vitro study on the human cervical spine, where for an applied lateral shear force the largest coupled motion was not lateral bending but axial rotation. In the more flexible neck of the barn owl, the upper region of the cervical vertebrae showed high H-roll and H-yaw capabilities when manually moved, but the lower cervical spine was mainly restricted to the H-roll, which then leads to lateral bending [16]. In human in vivo fluoroscopic evaluations, this effect was shown for the upper cervical spine but not for the lower areas. These differences could be explained by the effect intact soft tissues have in in vivo movement [41].

Significant correlations among yaw, pitch, and roll angles were observed at all eight CVJs across the four horses and movement directions with CVJ6 showing the lowest number of significant correlations, probably associated with the fact that its range of motion for pitch and yaw was lowest of all CVJs. Comparing locations, correlations at CVJ7 were most often significantly different from other locations, indicating distinct intersegmental behaviour at this location. Notably, CVJ1 demonstrated complete consistency, with yaw pitch and roll significantly correlated in all overall movements, highlighting its potentially critical role in coordinated cervical movement.

A review comparing studies on coupling of motions in the human cervical spine [42] noted greater variability in results for the upper cervical spine (CVJ1, CVJ2) compared to the lower cervical spine, where findings were more consistent. Over the large number of studies, variability was attributed to differences in measurement devices, movement initiation, and the distinction between in vivo and in vitro conditions. Such variability would be expected as well if future studies were to investigate motion coupling in the equine cervical spine in a different set up or even in vivo. Furthermore, more similar in vitro methodologies have been employed in other studies [20,43] where isolated human cervical specimens were used, with lower vertebrae fixated and upper vertebrae loaded with shear forces via pneumatic cylinders. These studies found that coupled axial rotation was more prevalent than lateral bending, and greater flexibility was observed in coupled flexion compared to coupled extension. This is not dissimilar to the results of the present study, where roll correlates more than yaw, and during overall flexion, slightly more variation in angular motion was noted than during extension.

In contrast, a human in vivo MRI study during lateral bending [18] demonstrated coupled axial rotation contralateral to the bending direction in the upper cervical levels but ipsilateral in the lower levels, with minimal coupled flexion–extension motion observed at all levels. Similarly, using the same setup, another study looking at the human spine during axial rotation [19] observed coupled lateral bending and axial rotation in the contralateral direction for CVJ1 and 2. These comparisons highlight the complexities of motion coupling patterns beyond species, conditions (in vivo vs. in vitro), and specific experimental setups.

In humans, the behaviour of coupling patterns depends on the initiation of movement, the posture of the spine and head, and any segmental pathology [20]. In vivo, this can also be explained by redundancy or abundance, whereby different motion strategies may achieve the same global movement depending on context. While this concept may similarly apply to horses, important anatomical differences must be considered. In contrast to the upright human spine, the equine cervical spine is horizontally aligned and supports the weight and mobility of the head at quadrupedal stance. Unlike in humans, where cervical motion is more evenly distributed across the vertebral segments [44], horses exhibit high mobility in the cranial cervical spine, functionally compensating for the more constrained motion within the caudal cervical spine. This highlights species-specific differences based on posture, joint morphology, and locomotor demands.

The experimental design of this study presents several limitations that warrant careful consideration when interpreting the findings. Notably, the absence of additional imaging—such as radiography, CT, or MRI—of the specimens represents a key constraint, as it precluded the integration of our results with diagnostic imaging data; an aspect that could have provided valuable insights. Moreover, the use of CT and/or MRI might have revealed pathologies that were not evident during gross pathological examination post-experiment, thereby offering complementary information to enhance the study’s outcomes.

Previous in vitro investigations of spinal movement in horses have employed various methodological approaches, including the use of isolated cervical spines or spinal segments subjected to independent movement [23,24,25,45,46], as well as partially dissected cervical specimens positioned laterally for measurement [47,48]. In the present study also, partially dissected specimens were used. Even though the ligamentous structures, most importantly the nuchal ligament, remained intact, the removal of the outer muscles is expected to have a relevant effect on the movement of the remaining structures, as is shown for the thoracolumbar spine of the goat [49]. One notable study utilised isolated equine cervical spines with the caudal portion secured in a vice while allowing manual manipulation of the free cranial end [22]. In contrast to these approaches, the present study employed a suspension system whereby a steel bolt was inserted through the base of the skull to achieve a neutral head and neck posture within a large wooden frame. While this methodology allowed for specimen suspension and positioning, the potential influence of this suspension system on the obtained results remains unquantified—a limitation not present in the fixed positioning methods employed in previous studies [22,47,48]. The incorporation of a pulley and rope system in our design provided some degree of flexibility and manoeuvrability, distinguishing our approach from the more rigid fixation methods reported in earlier investigations [23,24,25,45,46].

The present study’s methodology employed manual manipulation of the four horse cadaver specimens via a tightly fitted headcollar, a notable departure from prior mechanical or motorised testing paradigms [23,25,45,46]. All movements were performed by a single operator in consistent sequence and manner across specimens, with deliberate efforts to replicate natural physiological motion within plausible—not maximal—range of motion (ROM) limits of live horses [26,27]. While this approach achieved controlled, realistic positioning, the suspension of the skull inherently constrained the achievable ROM, introducing an unquantifiable influence on the observed correlations between yaw, pitch, and roll parameters.

Although the sample size was limited to four specimens, the observed variation in angular motion at the CVJs across these cadavers is argued to approximate the minimum variability that might be expected in a larger population. This assertion is grounded in the standardised methodology and the controlled, submaximal ROM employed, which likely mitigated extreme positional outliers and focused on biomechanically representative motions. Nonetheless, the small sample size of four horses remains a methodological constraint, as it precludes definitive conclusions about broader population-level variability.

Like in this present study, the use of frozen and thawed tissues is a common practice for experiments investigating the biomechanical properties of specimens. If performed with appropriate storage and care, even after multiple freeze and thaw cycles and a longer storage time, there is little to no effect on structural and mechanical properties, as has been shown on numerous occasions [50,51,52].

## 5. Conclusions

Clinically, knowledge of motion characteristics and their coupling at each CVJ of the horse provides valuable insight for diagnosis and therapeutic intervention. Abnormal motion patterns or unexpected coupling behaviours in specific segments may indicate localised pathology or biomechanical dysfunction. The study revealed strong motion coupling across all cervical vertebral joints (CVJs). However, the distribution of angular motion among individual joints varied considerably between horses, showing an abundance of movement possibilities. Future research should include larger and more diverse samples, including horses with cervical abnormalities, to improve clinical applicability.

## Figures and Tables

**Table 1:** Details of the donor horses used in this study.

	Breed	Sex	Age (yrs)	Reason for Euthanasia
Horse A	Warmblood	Gelding	13	Tumour conchae/sinus
Horse B	Unknown	Mare	6	Colic
Horse C	German Riding Pony	Mare	24	Spleen rupture
Horse D	Hanoverian	Gelding	10	Fracture third phalanx

## Data Availability

The raw data supporting the conclusions of this article will be made available by the authors on request.

## Abbreviations

The following abbreviations are used in this manuscript:

PCC	Pearson correlation coefficient
C1–C7	Cervical vertebra one to seven
CT	Computed tomography
MRI	Magnetic resonance imaging
T1	Thoracic vertebra one
H_y/p/r	Human yaw/pitch/roll
CVJ	Cervical vertebral joint
CVJs	Cervical vertebral joints (plural)
ROM	Range of motion

## Appendix A

Listed are the muscles removed from the specimens before movement analysis.

**Table A1:** Layers of muscles removed; nomenclature and grouping according to Nickel et al. and König and Liebich [53,54].

Musculi Capitis-Muscles for Specialised Movement of the Head	Musculi Colli-Long Muscles of the Neck	Musculi Dorsi-Long Muscles of the Back	Musculi Thoracis-Long Muscles of the Thorax
M. rectus capitis dorsalis major et minor	Mm. scaleni	M. trapezius	Mm. serrati dorsales et ventrales
M. rectus capitis lateralis et ventralis	M. splenius cervicis et capitis	M. rhomboideus cervicis et thoracis	M. transversus thoracis
M. obliquus capitis cranialis et caudalis	Mm. hyoidei	M. latissimus dorsi	M. rectus thoracis
M. longus capitis	M. sternocephalicus	Mm. pectorales	
	M. brachiocephalicus	M. iliocostalis thoracis	
	M. omotransversarius	M. longissimus atlantis, capitis, cervicis et thoracis	
	M. sternomandibularis	M. spinalis thoracis et cervicis	
		M. semispinalis capitis	
